# A Quantitative Evaluation of Global, Rule-Based Explanations of Post-Hoc, Model Agnostic Methods

**DOI:** 10.3389/frai.2021.717899

**Published:** 2021-11-03

**Authors:** Giulia Vilone, Luca Longo

**Affiliations:** Artificial Intelligence Cognitive Load Research Lab, Applied Intelligence Research Centre, School of Computer Science, Technological University Dublin, Dublin, Ireland

**Keywords:** explainable artificial intelligence, rule extraction, method comparison and evaluation, metrics of explainability, method automatic ranking

## Abstract

Understanding the inferences of data-driven, machine-learned models can be seen as a process that discloses the relationships between their input and output. These relationships consist and can be represented as a set of inference rules. However, the models usually do not explicit these rules to their end-users who, subsequently, perceive them as black-boxes and might not trust their predictions. Therefore, scholars have proposed several methods for extracting rules from data-driven machine-learned models to explain their logic. However, limited work exists on the evaluation and comparison of these methods. This study proposes a novel comparative approach to evaluate and compare the rulesets produced by five model-agnostic, post-hoc rule extractors by employing eight quantitative metrics. Eventually, the Friedman test was employed to check whether a method consistently performed better than the others, in terms of the selected metrics, and could be considered superior. Findings demonstrate that these metrics do not provide sufficient evidence to identify superior methods over the others. However, when used together, these metrics form a tool, applicable to every rule-extraction method and machine-learned models, that is, suitable to highlight the strengths and weaknesses of the rule-extractors in various applications in an objective and straightforward manner, without any human interventions. Thus, they are capable of successfully modelling distinctively aspects of explainability, providing to researchers and practitioners vital insights on what a model has learned during its training process and how it makes its predictions.

## 1 Introduction

Explainable Artificial Intelligence (XAI) has become a fundamental sub-field of Artificial Intelligence (AI). Its ultimate goal is to develop methods and techniques to produce data-driven machine-learned models with high accuracy and a high degree of explainability. In this context, explainability corresponds to the degree of transparency and understandability of the inner functioning of a model, and its inferences, as perceived by end-users. The availability of “big-data” and advances in computational processing have pushed machine and deep learning to a new high. This has led to the fast development of new and accurate models in a variety of domains. Unfortunately, most of these models are regarded as “black-boxes” due to their underlying complex structures that are unintelligible to end-users, thus requiring an explanation. As a consequence, several methods have emerged to extract information from trained models by trying to re-trace their inferential process in automatic ways (Došilović et al., 2018; Rizzo and Longo, 2018b). Some of these methods are model agnostic, meaning that, theoretically, they are suitable for any learning algorithms that can be wrapped with a layer of explanations. A subgroup of these methods extracts a set of rules that mimics the inferential process of the underlying machine-learned model (Došilović et al., 2018). The extracted rules can be in conflict with the expert domain knowledge pre-training of the model, thus perplexing the consumers of such tools. However, it must be considered that such rules aim at correctly representing the functioning of the black-box, thus the relationships between the independent variables of the input data with its dependent variable. Therefore, this conflict can be an essential signal of an issue occurring in the black-box model. Recent studies have attempted to solve these issues by integrating symbolic representations of knowledge with machine-learned models and producing explanations in the form of symbolic rules (Besold and Kühnberger, 2015). However, these rules are built upon a set of symbols not always and easily interpretable by lay humans. As a consequence, others attempted at building methods that generate if-then rules that should be more intuitive for humans, thus adding a meaningful descriptive layer to the underlying model (Letham et al., 2015). Nonetheless, little effort was devoted to assessing the degree of explainability of these rules objectively and quantitatively. From an in-depth analysis of the evaluation studies retrieved from the scientific literature, it was possible to notice that the authors took into account only a small number of notions and requirements to be satisfied by a rule-based explanation that aims to be intuitive and effective (Ferri et al., 2002; Freitas, 2006; Lakkaraju et al., 2016; Bologna and Hayashi, 2018; Ignatiev, 2020). An interpretable ruleset must contain a few short and concise rules whilst covering as many input instances as possible. Furthermore, none of these evaluation studies provides a tool to rank a set of rule-extraction methods according to the measurements of these notions to find out if one of them can be considered superior to the others.

This study aims at filling the above gap by proposing a framework for evaluating and comparing the degree of explainability of rule-based explanations automatically generated by XAI methods across eight metrics. These metrics assess various aspects of the explainability of automatically generated rulesets. If the rules score high according to all the metrics, they can provide valuable insights to researchers and practitioners about what a machine-learned model has learned from the input data during the training process. In other words, the rules can exhibit the model’s inner functioning and make explicit its logic. A set of objective, quantitative metrics also can form a versatile evaluation tool not bound to any particular rule-extraction method or model’s architecture. In detail, the research question tackled by this experiment is: “To what extent can a set of quantitative metrics be employed to assess and compare the degree of explainability of different if-then rule extraction methods?” The remainder of this manuscript is organised as follows. Section 2 summarises the strategies used by scholars to generate explanations of machine-learned models, with a focus on rule-extraction algorithms. Section 3 describes the design of this secondary research experiment and the metrics employed to evaluate and compare the degree of explainability of rulesets extracted by five XAI post-hoc model agnostic methods. Section 4 discusses the findings obtained from the experiment. Eventually, Section 5 emphasises the contribution to the body of knowledge and define future research directions.

## 2 Related Work

**FIGURE 1:**
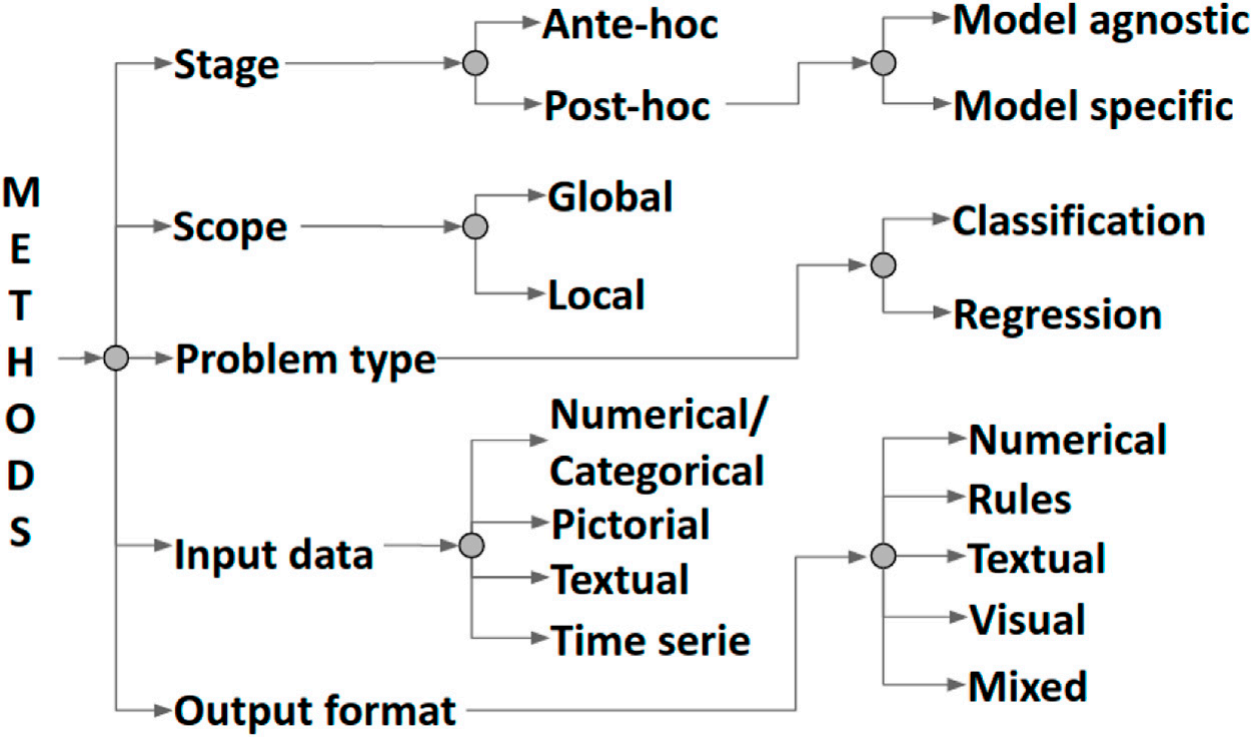
Classification of methods for explainability proposed in Vilone and Longo (2021a).

In the last few decades, researchers have tried to comprehend and explain the inner mechanics of data-driven machine-learned models in various ways (Longo et al., 2020; Guidotti et al., 2018). Consequently, several methods for explainability have been proposed over the years. These methods can be categorized along five dimensions (Vilone and Longo, 2021a), as shown in Figure 1. The first dimension is the scope of their explanation. The methods with a global scope attempt to make the entire inferential process of a model transparent and understandable as a whole. In contrast, the objective of local methods is to explain the inferential process around a specific input instance. Methods for explainability can generate explanations at two different stages. Ante-hoc methods tackle the explainability of a model from its implementation and during training. The goal is to make it naturally explainable while still trying to reach optimal accuracy and minimal error. Post-hoc methods, instead, keep a trained model unchanged and mimic or explain its behaviour by using an external explainer at testing time. The format of the input data of a model, which can be numerical/categorical, pictorial, textual, or a times series, can play an essential role in constructing a method for explainability because the logic followed by a learning technique can vary according to its inputs. The same can be said for the output format of the explanation itself, which can be numerical, rule-based, textual, visual, or mixed.

Explanations can have numerical formats as crisp values, vectors of numbers, matrices or tensors. These values provide a quantitative estimate of the relevance of the input features over the predictions of a model (Alain and Bengio, 2017; Kim et al., 2018). However, many people perceive numbers as dull and difficult to understand. Visual explanations illustrate, in a very appealing manner, the inner functioning of a model *via* graphical tools. Researchers have exploited several types of charts to make them as intuitive as possible and adapt the explanations to different input data and applications. For example, heat maps highlight the most influential parts of pictorial inputs to the target feature, such as areas of an image or a video, by using different colours (Ribeiro et al., 2016; Strobelt et al., 2018). Alternatively, data-flow graphs are employed to represent the inner structure of complex neural networks where each node represents either a layer or a neuron of the layer, and the edges the connections between layers of neurons (Wongsuphasawat et al., 2018). Textual explanations, consisting of natural language statements either written or orally uttered, are another intuitive form of explanation for humans. An example of these explanations is the phrase “This is a Brewer Blackbird because this is a blackbird with a white eye and long pointy black beak” produced by an explainer of an image classification model (Hendricks et al., 2018). Rules have a schematic, logical format, more structured than visual and textual explanations, but still intuitive for humans. They can be in the form of if-then statements with AND/OR operators, such as *IF* (*X*_1_ > 0.61) *AND* (*X*_2_ < 0.15) *THEN Class*_1_, and they are apt for reporting combinations of input features and their activation values (Fung et al., 2005; Bologna and Hayashi, 2017). Some rulesets structured as decision trees can be translated into logical formulas by combining split predicates along paths from inputs to predictions into logical conjunctions (AND) and all the paths related to an output class into logical disjunctions (OR) (Bride et al., 2018). Other rules employ symbolic logic, a formalized system of primitive symbols and their combinations such as (*Country* = *USA*) ∧ (28 < *Age* < = 37) → (*Salary* > 50*K*) where → logical operator joins the antecedents to their consequent (Ribeiro et al., 2018). Following this logic, rules can be implemented as fuzzy rules where one or more premises are linked to a consequent that can be true to a degree, instead of being entirely true or false. In these rules, antecedents and consequent are represented as fuzzy sets (Guillaume, 2001). The combination of fuzzy rules and learning algorithms can produce a powerful tool to perform reasoning and explain the inner logic of machine-learned models like neural networks (Palade et al., 2001). Such rules can be seen as arguments in the field of formal argumentation and reasoning. An argument, like a rule, presents a claim (or conclusion) that derives, soundly or not, from a set of premises. Arguments can be organised in a dialogical structure by employing attacks. Attacks represent conflicts between the premises and/or the conclusions of two or more arguments. Arguments and attacks form a graph of arguments that might possess an optimal explanatory capacity, suitable for representing the inner functioning of black-box data-driven models (Rizzo and Longo, 2018a,b). Eventually, some explanations employ one or more of the formats described so far (visual, textual, rules, numeric) to exploit their strengths and overcome their weaknesses. Some methods generate a mix of visual and textual explanations. For instance, Image Caption Generation with Attention Mechanism explains the logic of image classifiers by accompanying the classified images with captions highlighting in words their relevant parts (Xu et al., 2015). Other methods justify the prediction of a new input instance by identifying the most similar training samples or prototypes (Yeh et al., 2018).

Extracting explainable rules is one of the strategies followed by some global, post-hoc, model agnostic XAI methods to add a layer of explainability to a machine-learned model. Rules are built by approximating a model to be explained with a ruleset having a higher degree of interpretability. The underlying assumption is that, as long as the approximation quality is good, the statistical properties of the complex model are reflected in the interpretable rulesets. These rule extraction methods exploit various techniques. Genetic Rule EXtraction (G-REX) uses genetic algorithms to extract if-then rules with AND/OR operators (Johansson et al., 2004b,a). GLocalX is also based on a genetic algorithm to produce local rules explaining the prediction made by a classifier on each input instance (Guidotti et al., 2019; Setzu et al., 2021). The rules explicit both the factual reasons beyond the model’s logic and a set of counterfactuals highlighting which changes to the instance features lead to a different outcome. The local rules are hierarchically aggregated into a ruleset that covers the entire input space and represents a global explanation of the underlying model. Anchor generates if-then rules highlighting the “anchors” of an input dataset (Ribeiro et al., 2018). These are the features of a dataset that are sufficient for a classifier to make a prediction. For instance, the words “not bad” often appears in statements expressing a positive sentiment, thus can be considered anchors in sentiment analyses. Anchor uses two algorithms to identify the candidate rules with the highest estimated precision over a dataset. Precision is computed as the fraction of correct predictions. The first algorithm, a bottom-up formation of anchors, starts from an empty ruleset and iteratively adds a rule for each feature predicate. The second one instead consists of a beam-search for anchors. It starts from a set containing all the possible candidate rules and then selects the most precise ones. Model Extraction (Bastani et al., 2017) and Partition Aware Local Model (PALM) (Krishnan and Wu, 2017) approximates complex models with decision trees whose structure can be easily examined by end-users to determine if the rules match intuition. Model Extraction uses the Classification and Regression Trees algorithm (CART) and trains the extracted decision trees over a mixture of Gaussian distributions fitted to the input data using expectation maximisation. PALM consists of a two-part surrogate model: a meta-model, constrained to be a decision tree, partitioning the training data, and a set of sub-models fitting the patterns in the subset of data within each partition. Mimic Rule Explanation (MRE) selects a set of prototypes representing the whole input space and records the output class assigned to them by a model (Asano and Chun, 2021). Then, it perturbs each prototype to find out the maximum surrounding region where the predicted class remains unchanged. The resulting ruleset consists of the Cartesian product of finite intervals limiting these regions.

Along with research focused on the development of rule extraction methods, another branch of research in XAI is devoted to evaluating their degree of explainability. Scholars have identified a few attributes and notions that affect the degree of explainability of a ruleset (Bologna and Hayashi, 2018; Ferri et al., 2002; Freitas, 1999, 2006; Ignatiev, 2020; Lakkaraju et al., 2016; Vilone and Longo, 2021b). Rule size is one of such attributes, and it refers to the number of instances satisfied by a rule. Usually, researchers aim to extract rules that cover a large portion of the input data and support the discovery of new natural principles. Nonetheless, sometimes small rules might capture exceptions occurring in the data that can interest scientists. Furthermore, it might be more difficult, but more interesting, to discover rules aimed at predicting minority classes when the imbalance of class distributions occurs in the data. Attribute costs represent the effort to collect or get access to the actual value of an attribute of the data. For instance, it is easy to assess the gender of a patient, whereas other health-related parameters can require an expensive investigation (Freitas, 1999). Rules that use only attributes based on easily accessible data are more appealing as they help keep the costs of an experiment low. In some domains of application, such as medicine, it is vital to assess the misclassification costs of a ruleset because the erroneous classification of an instance might have a significant impact, not only in monetary terms but also in numbers of human lives. To be quantified, all these costs require integrating the user’s domain knowledge which is a manual and time-consuming process. Luckily, scholars have identified several other attributes of explainability that can be assessed objectively as they need the information provided by the input and output data without relying on domain knowledge (Alonso et al., 2018; Abdul et al., 2018; Miller, 2019; Lakkaraju et al., 2016). The symbolic rules extracted by Discretized Interpretable Multilayer Perceptron (DIMLP) from ensembles of artificial neural networks are compared with boosted shallow trees and support vector machines. The comparison is made by computing the complexity, measured as the total number of rule antecedents per ruleset, the prediction accuracy and the fidelity of the rulesets (Bologna and Hayashi, 2018) (see Table 3 for the definition of fidelity). Four criteria, translated into five metrics, have been proposed to evaluate the degree of explainability of if-then rules automatically generated by a method called Interpretable Decision Sets (Lakkaraju et al., 2016, 2017). According to these criteria, rulesets can be considered interpretable if (I) its rules describe non-overlapping areas of the feature space, (II) its rules cover most (ideally all) data points and (III) most (ideally all) the classes in the data, and (VI) the ruleset comprises a small number of concise rules. The five resulting metrics are (I) fraction overlap which captures the extent of overlap between every pair of rules of a ruleset, (II) fraction uncovered which computes the fraction of the input dataset not covered by any rule, (III) average rule length which captures the average number of antecedents of each rule, (IV) number of rules which is the cardinality of the ruleset, and (V) fraction of classes which measures the fraction of the output classes in the data predicted by at least one rule. The second metric can be considered as the inverse of completeness, which is a requirement identified in other studies, and it was thus preferred to the fraction uncovered (Cui et al., 2019). Beyond these attributes and metrics, scholars proposed other quantitative validation factors that must be fulfilled by every type of explanation automatically generated by an XAI method. These include the correctness of a ruleset, measured as the portion of the dataset correctly classified by rules, its fidelity to the predictions of the model and robustness, understood as the capacity to withstand small perturbations of the inputs that do not affect the predictions of the model, thus should not affect the predictions of the ruleset. The rules should also be minimal, meaning that they could not be discarded without compromising the prediction accuracy of the ruleset. These metrics must be maximised to generate trustable explanations (Ignatiev, 2020). Fidelity, correctness and the average number of rules were used to compare the degree of explainability of three rulesets automatically extracted from machine-learned models in (Veerappa et al., 2021). The correctness of these rulesets was assessed with the *F*_1_ − *scores*. Despite these efforts to propose evaluation approaches and metrics for explainability, there are still critical gaps in this area of XAI. Firstly, there is no general consensus among scholars on when an explanation can be considered as such. Thus, it is impossible to say with certainty if a machine-generated ruleset represents a viable solution to the XAI quest. Secondly, it is unknown which salient properties a ruleset must possess to be effective and understandable by end-users. This study aims at filling these gaps by proposing an evaluation framework for rule-extraction methods for explainability. In accordance with Adadi and Berrada (2018), scholars have produced enough material to make this objective achievable.

## 3 Design and Research Methods

The subset of methods for explainability generating if-then rules from the inferences of machine-learned models is quite large, so it was necessary to narrow it down by adding the following three inclusion criteria:

1. The methods must be model-agnostic, meaning that they do not consider the internal components of a model such as weights or structural information. Therefore they can be applied to any black-box model. This limits the choice of the post-hoc methods as the ante-hoc ones are inherently model-specific.
2. The extracted rules must represent a global explanation of a black-box model.
3. The output ruleset must be comprised of if-then rules or rules that can be translated into this format.

Five rule-extraction methods fulfil these criteria, namely C4.5Rule-PANE, REFNE, RxREN, RxNCM, and TREPAN (described in section 3.2). Their respective algorithms are described in the following section and summarised with the pseudo-codes in the first section of the Supplementary Material.

### 3.1 The Experiment Design

**FIGURE 2:**
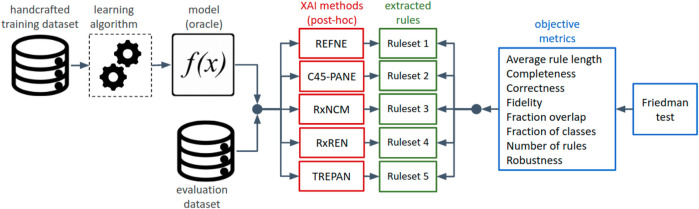
Diagrammatic view of the design of the experiment for evaluating and comparing the explainability of rule-sets, automatically extracted from neural networks by different methods for explainability.

To answer the research question, the experiment was designed as shown in the diagram of Figure 2. A model, which is the output of a learning algorithm (in this study, feed-forward neural networks) trained on an input dataset, and an evaluation dataset were fed into the five methods for explainability under analysis. Each method extracted a set of if-then rules whose degree of explainability was assessed with eight objective and quantitative metrics. This process was repeated over 15 datasets and their neural networks.

### 3.2 Rule Extraction Methods

Rule Extraction From Neural Network Ensemble (REFNE) was developed to extract symbolic rules from ensembles of trained neural networks, but it can be easily applied also to other learning approaches (Zhou et al., 2003). The REFNE algorithm replaces the original labels of a training dataset with those predicted by the trained model. Subsequently, REFNE queries the underlying model to generate new instances to be added to the training dataset. These instances are created by randomly selecting the value of each feature across its range, so diverse input patterns can appear as frequently as the data allow and the entire input space is covered as much as possible. According to the authors, it is enough to double the size of the training set to generate accurate rules mimicking the underlying trained model. To extract rules, REFNE randomly selects a categorical feature and checks if there is a value such that all the instances possessing it fall into the same class. If this condition is satisfied, a rule is created with the value as antecedent. Otherwise, another categorical feature is selected, and the same process is repeated. If no rules have been created after examining all the single features, the algorithm performs a pairwise analysis of the categorical features by combining their values and checking if it is possible to create new rules with two antecedents. The algorithm keeps increasing the number of combined attributes. Rules are limited to only three antecedents because, according to the authors, longer rules are unintelligible to humans (Zhou et al., 2003). When all the categorical features have been examined, the continuous ones are discretised and considered as new categorical features. The process terminates when no more rules can be created, or all the input instances have been covered. In the original design, the discretisation process is carried out with the ChiMerge algorithm. However, in this study, the ChiMerge algorithm was replaced with a less compute-intensive discretisation process, as described in the second section of the Supplementary Material. Finally, a new rule is added to the output ruleset only if its fidelity (as defined in Table 3) to the underlying model is above a user-defined threshold.

C4.5Rule-PANE is an alternative method to REFNE, and it also extracts if-then rules from ensembles of neural networks (Zhou and Jiang, 2003). It uses the C4.5 rule induction algorithm to construct a ruleset that mimics the inferential process of the underlying model from a synthetic dataset built by (I) replacing the original labels of the training dataset with those predicted by the underlying model and (II) creating new instances generated by randomly selecting the values of the input features from their ranges and feeding them into the model.

Similarly, TREPAN induces a decision tree by querying the underlying trained model to determine the output class of each instance (Craven and Shavlik, 1994; Craven and Shavlik, 1996), thus replacing the original labels. Subsequently, it splits each node of the tree by using the gain ratio criterion. To ensure a minimum sample of instances available at each node, TREPAN queries the underlying network to generate new samples. The previously selected splits that lie on the path, from the root to the node under analysis, are considered constraints. TREPAN generates a complete instance by randomly selecting values for each feature while satisfying the constraints. To model each feature’s marginal distribution, TREPAN uses frequency counts for discrete features and kernel density estimation method to model continuous features. TREPAN expands trees using a best-first expansion algorithm where the best node has the greatest potential to increase the fidelity of the extracted tree to the network. To evaluate a node *n*, TREPAN uses the function *reach*(*n*) × [1 − *fidelity*(*n*)] where *reach*(*n*) is the estimated fraction of instances that reach *n* and *fidelity*(*n*) is the estimated fidelity of the tree to the network for those instances. The split values of each node are selected using *m*-of*n* expressions constructed with a hill-climbing search process that begins by selecting the best binary split. Then, it employs a beam search method, with beam width as a user-defined parameter, to find the best *m*-of*n* split according to the gain-ration criterion, utilised as the split evaluation function. TREPAN uses two independent criteria to decide when to stop creating new nodes: 1) the tree has reached the maximum tree size, which is a user-specified parameter (in this experiment, this limit was set equal to 15 to limit the computational time), and 2) a node is set as a leaf if, with high probability, the node covers only instances belonging to the same class.

Rule Extraction by Reverse Engineering (RxREN) (Augasta and Kathirvalavakumar, 2012) is based on a recursive algorithm to generate hierarchical if-then rules. It relies on a reverse engineering technique to trace back relevant input features that lead to the final results. Then, it prunes the insignificant input neurons whilst requiring that the accuracy of the pruned model decreases at most by 1% from the original model. Next, it computes the data ranges of each significant neuron in each output class by iteratively removing one input feature at a time and measuring the impact on the number of misclassified instances. This process can be seen as a feature selection approach, and it is easily applicable to other architectures. Rule Extraction from Neural Network using Classified and Misclassified data (RxNCM) (Biswas et al., 2017) is a modification of RxREN. It includes the input instances correctly classified in the range determination process, besides the misclassified ones exclusively considered by RxREN.

### 3.3 Dataset

These methods were tested on 15 public datasets (listed in Table 1) retrieved from the UCI Machine Learning Repository^1^ and briefly described in the next paragraph. The datasets were selected according to the following criteria: (I) all the datasets must be handcrafted, meaning that their features were manually engineered by humans and not the output of an algorithm, (II) they must contain enough instances to avoid the curse of dimensionality, meaning too many features for too few instances. A typical rule of thumb is that there must be five training samples for each feature (Theodoridis and Koutroumbas, 2009). To be on the safe side, the number of instances must be at least in the order of ten thousand, and (III) the dependent variable is categorical, ideally with more than two target classes, whereas the independent variables are both continuous and categorical predictors.

**TABLE 1:** Properties of the selected datasets.

Dataset	Total instances	No. of input features	No. of continuous (categorical) features	No. of classes
Adult	48,842	14	6 (8)	2
Avila	20,867	10	10 (0)	12
Bank marketing	45,207	20	11 (9)	2
Chess	28,056	6	3 (3)	18
Connect 4	67,557	42	0 (42)	3
Cover type	581,012	54	10 (44)	7
Credit card default	30,000	23	20 (3)	2
EEG eye state	14,980	14	14 (0)	2
HTRU	17,898	8	8 (0)	2
Letter recognition	20,000	16	16 (0)	26
Occupancy	12,417	5	5 (0)	2
Online shopper intention	12,330	17	14 (3)	2
Person activity	164,860	4	3 (1)	11
Shuttle	58,000	9	9 (0)	7
Skin	245,057	3	3 (0)	2

The Adult database is based on the 1994 US Census, and it was designed to train models to predict whether a person makes or not over $50K. The Avila dataset contains information about 800 images of the “Avila Bible,” a Latin copy of the whole Bible produced during the XII century by 12 Italian and Spanish copyists individuated from a palaeographic analysis of the manuscript. Thus, the prediction task is to associate each image with the copyist who drew it. The Bank marketing dataset collects data related to a marketing campaign carried out by Portuguese banks. The output variable registered whether the customer subscribed to a term deposit. The Chess dataset contains the coordinates of the White King and Rook and the Black King and Knight, whereas the output variable records the optimal depth-of-win for the White in 0–16 moves. Otherwise, it assigns a draw. Similarly, the Connect-4 dataset reports all the 8-ply positions in the connect-4 game in which none of the players has won yet, and the next move is not forced. The output variable is the theoretical outcome of the game for the first player (win, loss, draw). The Cover Type dataset contains 12 cartographic measures of 4 wilderness areas of the Roosevelt National Forest of northern Colorado to predict the seven forest cover types, depending on the major trees species in these areas. The Credit Card Default dataset was designed for classification models that predict whether Taiwanese clients will suffer failure to repay their credit card debts. The EEG Eye State dataset reports the combined information of continuous electroencephalogram (EEG) measurements and the eye state (either open or close), the target variable. The High Time Resolution Universe (HTRU) dataset describes the physical properties of stars to determine whether they are pulsars or not. The objective of the Letter Recognition dataset is to identify each of the 26 capital letters of the English alphabet, displayed on black-white images, from 16 primitive numerical attributes (statistical moments and edge counts) calculated over the pixels. The Occupancy dataset was created for the binary classification problem of determining whether a room is occupied from environmental information such as temperature, humidity, light ,and CO_2_. Online Shopper Intention records thousands of sessions on e-commerce websites. The negative output class represents customers who did not buy anything, whilst the positive class represents sessions that ended with purchasing the searched item. The Person Activity dataset contains data related to 11 physical activities, such as walking or sitting, recorded from people wearing four ankles, belts, and chest sensors. The Shuttle dataset reports nine numerical attributes of a shuttle, and it was designed for the classification task of determining its flight stage, such as if it is flying at a high altitude or if it is either taking off or landing. Finally, the Skin dataset contains information on the three colour channels of images, some of which contain faces of various ages, races, and genders. The scope is to determine which images represent people’s faces.

Before being fed into a machine-learned model, the datasets were pre-processed to handle missing data and remove correlated variables, which can cause issues in the training process of the models. None of the selected datasets contains missing data, so no action was required. However, some input features had to be discarded because they could not be considered valid predictors as they did not represent discriminative attributes. These features are:

• “fnlwgt” of the Adult dataset, which contains the statistical weights assessing how many US citizens are represented by each subject
• The client “ID” from the Credit Card Default dataset
• “Sequence name” (which correspond to the subject code), “timestamp” and “date” from the Person Activity dataset

Afterwards, a correlation analysis was performed on each dataset to detect pairs of highly correlated features. If this was the case, one of the two strongly correlated features was discarded to reduce multicollinearity risk. Firstly, a correlation matrix containing the Spearman’s rank correlation coefficients of each pair of input features was computed for each dataset. Unfortunately, there is no consensus on the thresholds between strong, moderate and weak correlations among the scientific community. In this study, the range of the absolute Spearman’s rank values, which is (0, 1), was split into three segments, where values in the range (0, 0.33) are considered weak, (0.33, 0.66) moderate and (0.66, 1) strong correlations. Secondly, it was necessary to find an objective way to decide which variable of each strongly correlated pair should be discarded. The best subset selection analysis was carried out to chose, among all the possible combinations of not-strongly-correlated variables, the combination that best fits the outcome variable (Hocking and Leslie, 1967). A linear regression model was built over each combination of the input variables strongly correlated with at least another variable (this analysis excluded those variables that do not show any strong correlations). These models were then sorted in descending order according to the *R* ^2^ value. The combination free from strong-correlated pair of variables and with the highest *R* ^2^ was selected. The best subset selection approach was chosen for its simplicity and because it requires little computational time and resources. Finally, some of the chosen datasets are unbalanced, meaning that their target variables have more instances in one specific class than the others. Due to this disparity, some learning algorithms might classify all the input instances into the majority class whilst ignoring the other minority classes. To avoid this issue, each dataset was split into a training and a validation dataset by using the stratified five-fold cross-validation technique to ensure that each class is represented with the same proportion as in the original dataset (Stone, 1974). Additionally, the Synthetic Minority Over-Sampling Technique (SMOTE) has been applied to the training datasets to up-sample the minority classes, thus giving each class the same chance to influence the resulting trained model (Chawla et al., 2002).

### 3.4 Models

**FIGURE 3:**
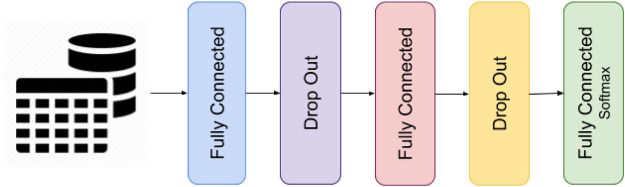
Architecture of the feed-forward neural networks.

The models trained on the 15 datasets were all feed-forward neural networks with two fully-connected hidden layers, both coupled with a dropout layer (see Figure 3). These networks were chosen to assess the feasibility of the proposed experiment. The number of nodes in the hidden layer, together with other hyperparameters of the networks, such as the node’s activation function, was determined by performing a grid search to reach the highest feasible prediction accuracy. Table 2 reports the list of the optimal values of the hyperparameters together with the prediction accuracy obtained on the 15 datasets. The loss function was set equal to the categorical cross-entropy since the networks were trained on categorical variables. Studies show that the categorical cross-entropy function has advantages over other loss functions, like those based over the squared-error (Kline and Berardi, 2005). The early stopping method was utilized to avoid overfitting during the training process by limiting the number of training epochs and stop the training process when the validation accuracy of the model did not improve for five epochs in a row. In any case, the number of training epochs could not exceed 1000. The dropout rates were varied in the range from 0 to 50% (in units of 10%), the batch size was searched among 1, 16, 32, 64, 128, and 256 whilst the number of neurons in each hidden layer (both layers have the same number of neurons) was selected by following the method proposed in (Doukim et al., 2010). In the first iteration, the number of hidden neurons was determined using the binary search mode. This number was selected from 1, 2, 4, 8, 16, 32, 64, and 128 and corresponded to the value with the highest prediction accuracy. Then, a sequential search was used in the neighbourhood of the previously selected value by increasing it by one unit to check if there is a further improvement in the prediction accuracy value. This second step was performed when the accuracy was lower than 80%. Each neural network was trained five times over the five training subsets extracted from each input dataset with the five-fold cross-validation technique. The network with the highest validation accuracy was selected and fed into the rule-extraction methods.

**TABLE 2:** Optimal hyperparameters of neural networks obtained through grid search procedure, grouped by dataset, and their resulting accuracies.

Model parameters	Dataset list				
	Adult	Avila	Bank	Chess	Connect 4
Optimizer	Adam	RMSprop	Adamax	SGD	SGD
Weight initialisation	Uniform	He-Uniform	Normal	Lecun-Uniform	He-Uniform
Activation function	Tanh	Relu	Softplus	Softplus	Softmax
Dropout rate	0%	0%	10%	0%	0%
Batch size	128	16	16	8	8
Hidden neurons	16	32	32	24	8
Accuracy	86.73%	94.87%	92.40%	77.07%	68.21%
(validation set)	(82.08%)	(83.88%)	(90.81%)	(34.40%)	(68.61%)
	Cover type	Credit card default	EEG eye states	HTRU	Letter recognition
Optimizer	NAdam	NAdam	Adadelta	RMSprop	RMSprop
Weight initialisation	Normal	Lecun-uniform	Glorot-uniform	Normal	Glorot-Uniform
Activation function	Hard sigmoid	Relu	Softsign	Softsign	Linear
Dropout rate	0%	0%	0%	20%	0%
Batch size	256	64	1	16	64
Hidden neurons	64	32	32	4	16
Accuracy	83.18%	54.44%	54.53%	93.72%	72.47%
(validation set)	(56.67%)	(74.70%)	(55.34%)	(97.77%)	(72.45%)
	Occupancy	Online shopper intention	Person activity	Shuttle	Skin
Optimizer	RMSprop	Nadam	RMSprop	RMSprop	Adamax
Weight initialisation	Zero	Normal	Lecun-Uniform	He-Uniform	He-Uniform
Activation function	Sigmoid	Tanh	Tanh	Softsign	Softsign
Dropout rate	30%	50%	0%	0%	40%
Batch size	64	64	256	32	16
Hidden neurons	2	8	256	4	3
Accuracy	90.49%	79.18%	56.20%	99.52%	90.26%
(validation set)	(99.24%)	(92.17%)	(43.87%)	(98.72%)	(98.23%)

### 3.5 Metrics

Eight metrics were selected to assess, in an objective and quantitative manner, the degree of explainability of the rulesets generated by C4.5Rule-PANE, REFNE, RxREN, RxNCM, and TREPAN from the neural networks trained on the 15 datasets. The objectivity is reached by excluding any human intervention or expert’s background knowledge in this evaluation process. Two of these metrics, number of rules and average rule length, are attributes of explainability and measure the syntactic simplicity of the rules. The ideal ruleset should minimise both of them in order to be easily interpreted and understood by end-users (Lakkaraju et al., 2016). Fraction of classes and fraction overlap enhance the clarity and coherence of the extracted rules. Whilst the fraction overlap should be minimised to avoid conflicts between the rules, the fraction of classes should be maximised to guarantee that all the target classes, even the minor ones, are considered. A ruleset must also score high in the remaining four metrics (completeness, correctness, fidelity, and robustness), which measure the agreement between the explanation method and the machine-learned model. This means that the ruleset can appropriately classify any input instances, it is faithful to the underlying model, and its inferences do not vary when inputs are slightly distorted by applying a Gaussian noise. These eight metrics can be easily measured from the inferences of the machine-learned models and the rulesets without requiring the integration of domain knowledge. Table 3 reports their definition and the formulas used to calculate them.

**TABLE 3:** Objective metrics to assess the explainability of rulesets.

Factor	Definition	Formula
Completeness	Ratio of input instances covered by rules (*c*) over total input instances (*N*) Cui et al. (2019)	$\frac{c}{N}$
Correctness	Ratio of input instances correctly classified by rules (*r*) over total input instances Ignatiev (2020)	$\;\frac{r}{N}$
Fidelity	Ratio of input instances on which the predictions of model and rules agree (*f*) over total instances Saad and Wunsch (2007)	$\frac{f}{N}$
Robustness	The persistence of methods to withstand small perturbations of the input (*δ*) that do not change the prediction of the model (*f* (*x*_*n*_)) Alvarez-Melis and Jaakkola (2018); Liu et al. (2017)	$\left[t\right]\frac{\sum_{n=1}^{N}f\left(x_{n}\right)-f\left(x_{n}+\delta \right)}{N}$
Number of rules	The cardinality of the ruleset (*A*) generated by the four methods under analysis Freitas (1999); García et al. (2009); Lakkaraju et al. (2016)	|*A*|
Average rule length	The average number of antecedents, connected with the AND operator, of the rules contained in each ruleset Lakkaraju et al. (2016); Wu et al. (2018). *a*_*i*_ represents the number of antecedents of the *i*th rule and *R* = |*A*| the number of rules	$\frac{\sum_{i=1}^{R}a_{i}}{R}$
Fraction of classes	Fraction of the output class labels in the data are predicted by at least one rule in a ruleset R . A rule *r* is represented by a tuple (*s*, *c*) where *s* is the set of antecedents and *c* is a class label. |C| represents the number of class labels Lakkaraju et al. (2016)	$\frac{1}{\left|C\right|}\sum_{c^{\prime }\leq C}1\left(\exists r=\left(s,c\right)\in R|c=c^{\prime }\right)$
Fraction overlap	The extent of overlap between every pair of rules of a ruleset. Given two rules *r*_*i*_ and *r*_*j*_, overlap is the set of data points that satisfy the conditions of both rules Lakkaraju et al. (2016)	$\frac{2}{R\left(R-1\right)}\sum_{r_{i},r_{j},i\leq j}\frac{overlap\left(r_{i},r_{j}\right)}{N}$

The final step of this scientific experiment consists of ranking the selected methods for explainability according to these eight metrics in an objective and automatic way. To be verified with a statistical test, the research hypothesis is that there are statistically significant differences in the degree of explainability of rulesets automatically extracted by the five rule-extraction methods. The Friedman test, a non-parametric statistical test designed to detect differences in treatments (the four methods for explainability) across multiple test attempts (the eight metrics), was applied to check whether any methods for explainability ranked consistently higher (or lower) according to the metrics of choice. The alternative hypothesis of the test is that there are significant differences in the results of the five methods; hence one of them can be ranked as the best. The Friedman test was chosen instead of ANOVA because it was not possible to fulfil the latter’s assumption on the distribution of the samples. Samples must come from normally distributed populations with equal standard deviations.

## 4 Results and Discussion

**FIGURE 4:**
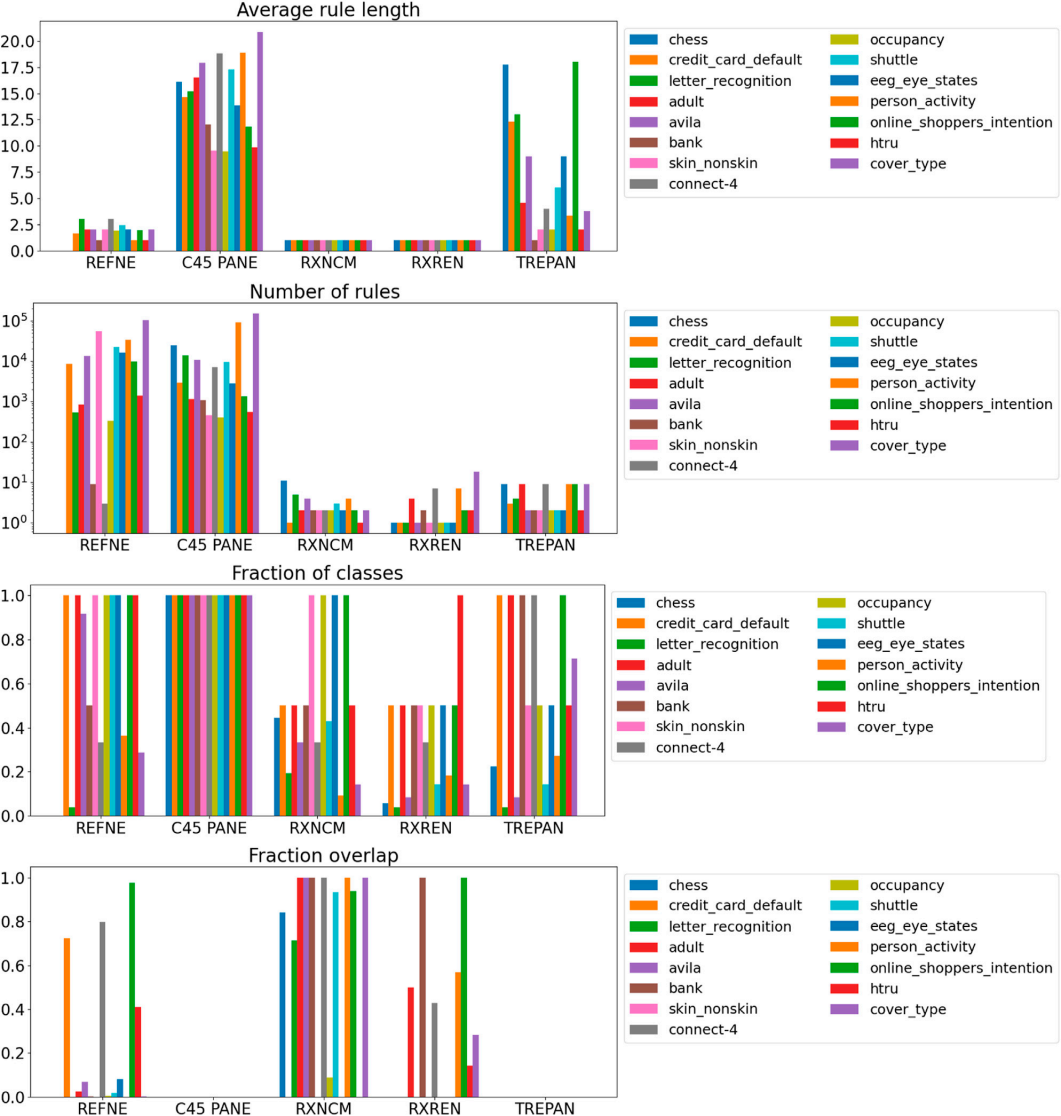
Quantitative measures of the degree of explainability of the rulesets automatically generated by 5 rule-extraction methods over 15 datasets, grouped by method.

**FIGURE 5:**
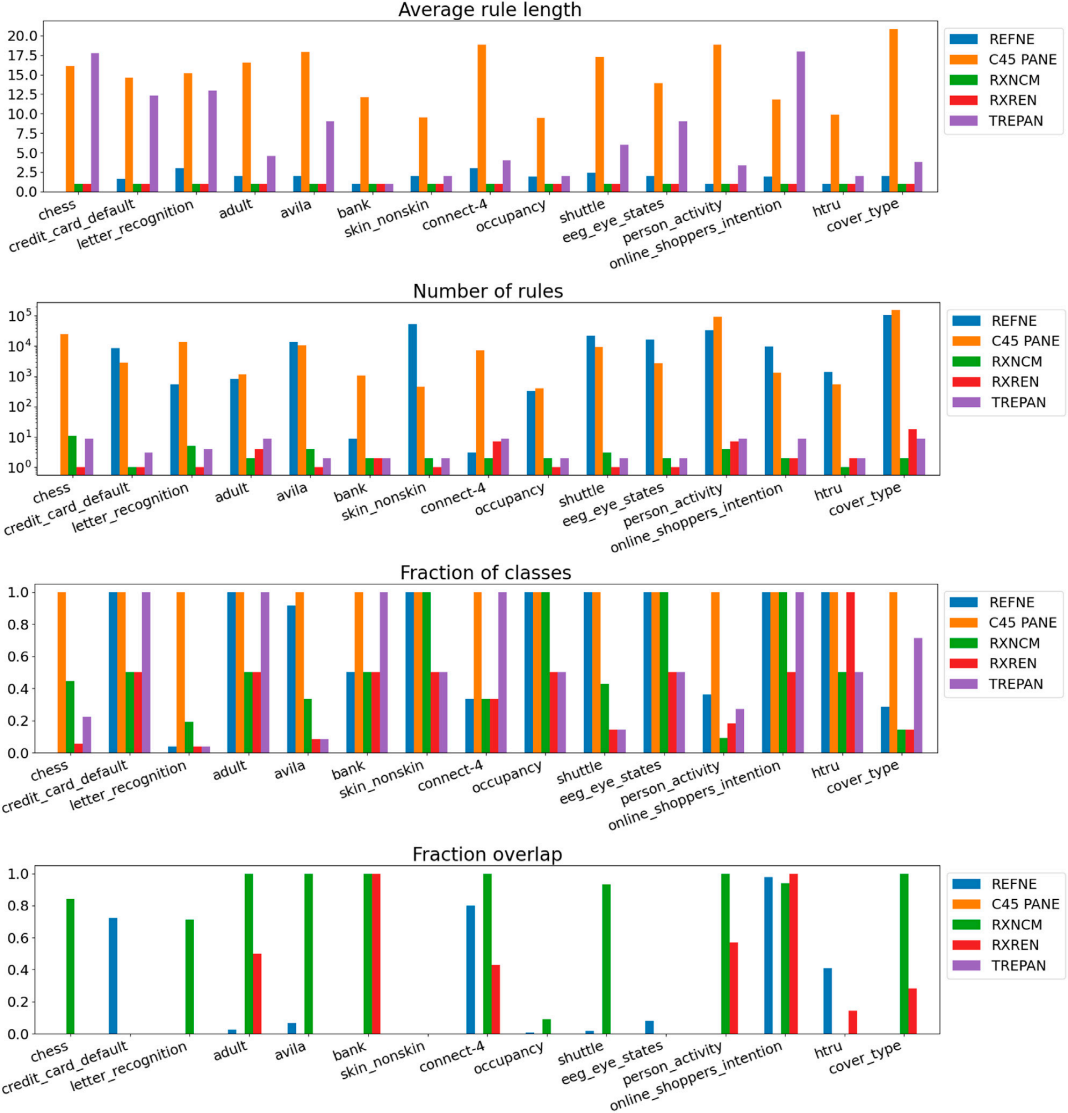
Quantitative measures of the degree of explainability of the rulesets automatically generated by 5 rule-extraction methods over 15 datasets, grouped by dataset.

**FIGURE 6:**
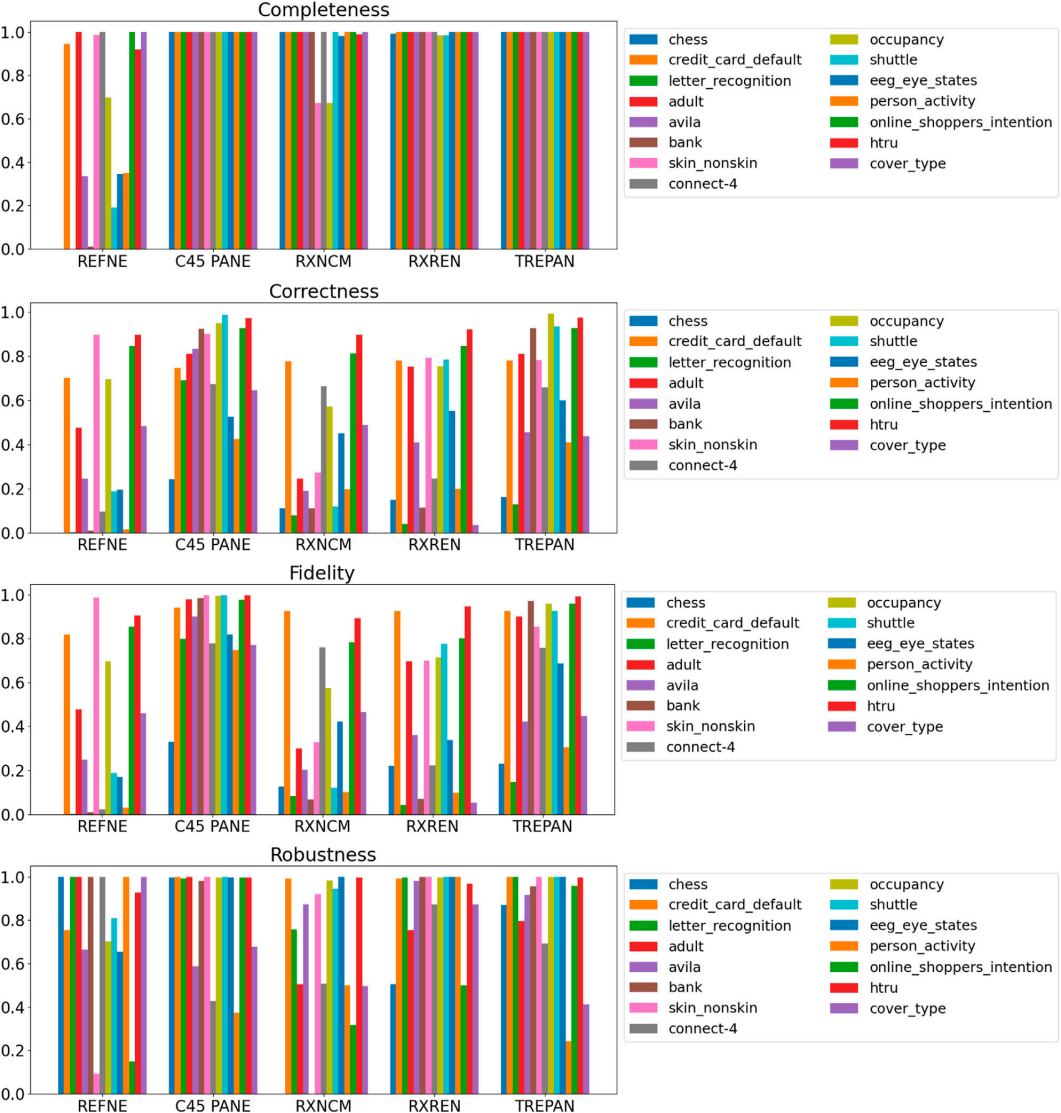
Quantitative measures of the degree of explainability of the rulesets automatically generated by 5 rule-extraction methods over 15 datasets, grouped by method.

**FIGURE 7:**
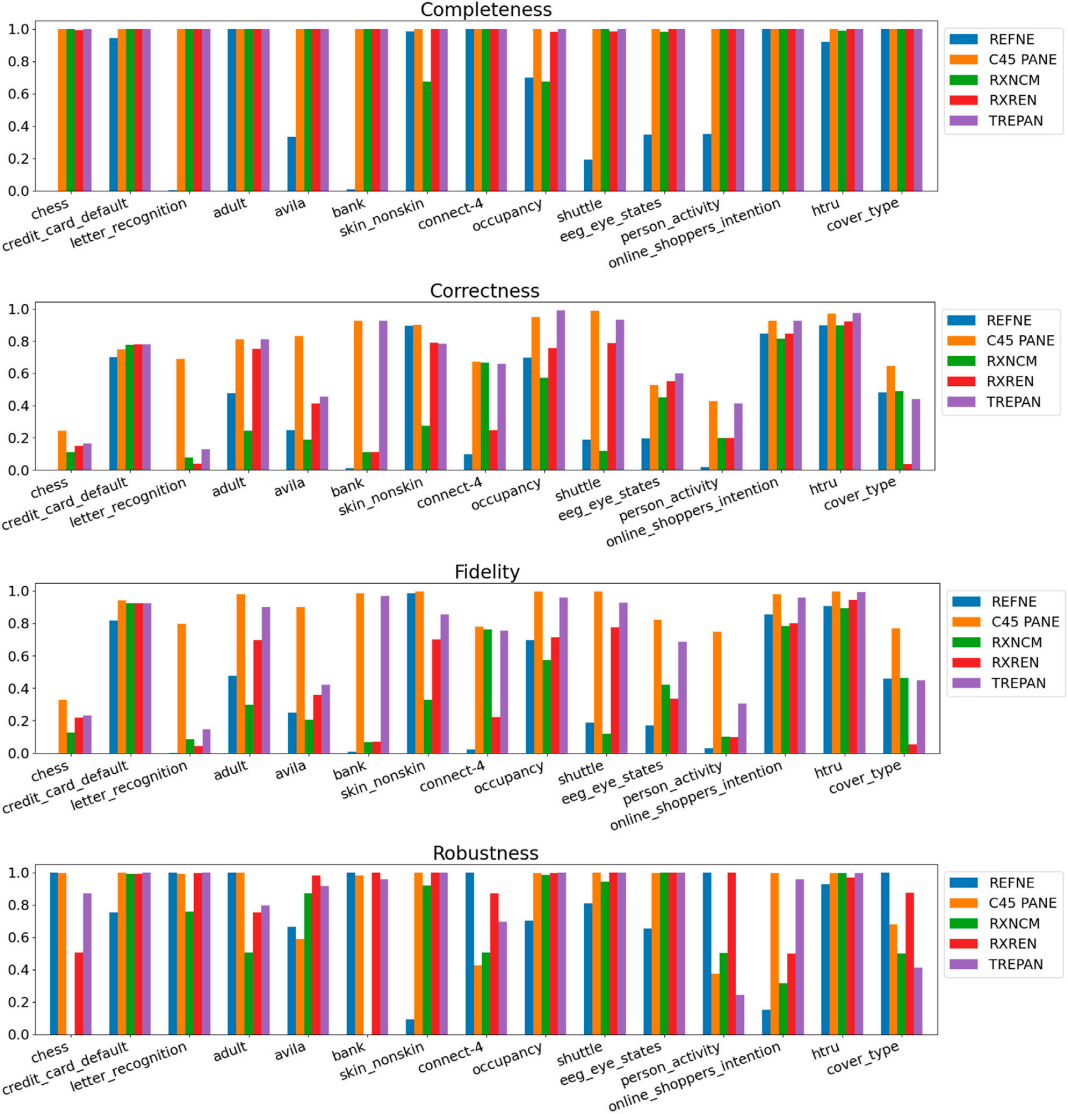
Quantitative measures of the degree of explainability of the rulesets automatically generated by 5 rule-extraction methods over 15 datasets, grouped by dataset.

**FIGURE 8:**
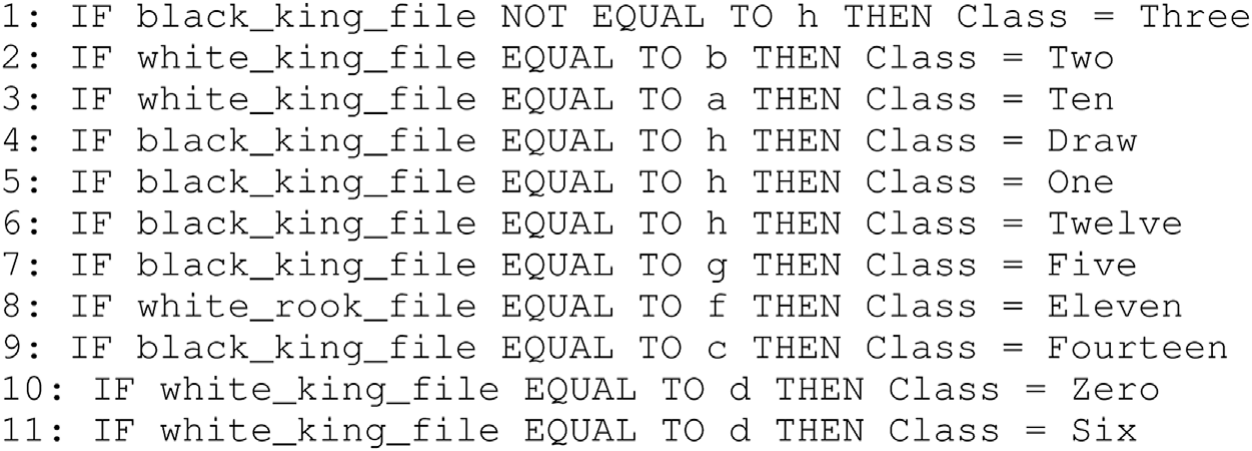
Example of ruleset automatically generated by a rule-extraction method. In this case, the ruleset was created by RxNCM from the Chess dataset.

Results related to the four metrics measuring the syntactic simplicity and coherence of the 15 machine-generated rulesets are presented in Table 4 and in Figures 4, 5 grouped respectively by method for explainability and dataset. Figures 6, 7 report the results of the other four metrics, namely completeness, correctness, fidelity and robustness. An example of a ruleset automatically generated by the rule extraction methods is shown in Figure 8.

**TABLE 4:** Quantitative measures of the degree of explainability of the rulesets automatically generated by 5 rule-extractor methods over 15 datasets according to 8 metrics.

Dataset	REFNE	C4.5-PANE	RxNCM	RxREN	TREPAN	REFNE	C4.5-PANE	RxNCM	RxREN	TREPAN
	Completeness					Correctness				
Adult	1.0	1.0	1.0	1.0	1.0	0.4748	**0.8112**	0.2441	0.7521	0.8097
Avila	0.3349	1.0	1.0	1.0	1.0	0.2453	**0.8323**	0.1888	0.4109	0.4547
Bank	0.0102	1.0	1.0	1.0	1.0	0.0102	0.9246	0.1107	0.1123	**0.9273**
Chess	0.0	1.0	1.0	0.9911	1.0	0.0	**0.2431**	0.1095	0.1495	0.1623
Connect 4	1.0	1.0	1.0	1.0	1.0	0.0955	**0.6734**	0.6641	0.2462	0.6586
Cover type	1.0	1.0	1.0	1.0	1.0	0.4835	**0.6448**	0.4882	0.0353	0.4387
Credit card default	0.9442	1.0	0.9994	1.0	1.0	0.7017	0.748	0.7783	**0.7787**	**0.7787**
EEG eye states	0.3455	1.0	0.9826	0.9997	1.0	0.1936	0.5267	0.451	0.5511	**0.6008**
HTRU	0.9184	1.0	0.9889	1.0	1.0	0.8966	0.9715	0.8975	0.9221	**0.9749**
Letter recognition	0.0015	1.0	0.999	1.0	1.0	0.0008	**0.6903**	0.0766	0.0395	0.1295
Occupancy	0.6989	1.0	0.6734	0.9835	1.0	0.6957	0.9485	0.5732	0.7548	**0.9928**
Online shopper intention	1.0	1.0	0.9986	1.0	1.0	0.8467	**0.9266**	0.8135	0.8455	0.9258
Person activity	0.3504	1.0	1.0	1.0	1.0	0.0157	**0.4251**	0.1966	0.1984	0.4109
Shuttle	0.1909	1.0	0.9996	0.9839	1.0	0.1873	**0.9872**	0.1185	0.7859	0.9339
Skin	0.9858	1.0	0.6736	0.9995	1.0	0.8958	**0.9019**	0.2738	0.792	0.7829
	**Fidelity**					**Robustness**				
Adult	0.4774	**0.9793**	0.2985	0.6972	0.8996	**1.0**	0.9996	0.5059	0.7551	0.7948
Avila	0.2492	**0.8991**	0.204	0.3603	0.4207	0.6644	0.5877	0.872	**0.9808**	0.9169
Bank	0.0102	**0.9849**	0.0687	0.0695	0.9701	**1.0**	0.9808	0.0	0.9999	0.9566
Chess	0.0	**0.3291**	0.1252	0.2202	0.2309	**1.0**	0.9968	0.0	0.5046	0.871
Connect 4	0.0226	**0.7786**	0.7609	0.2227	0.7565	**1.0**	0.4273	0.5067	0.8729	0.694
Cover type	0.4602	**0.7704**	0.4645	0.0522	0.448	**1.0**	0.6769	0.4982	0.8744	0.4128
Credit card default	0.8187	**0.9417**	0.925	0.925	0.925	0.756	0.9997	0.9916	0.9923	**1.0**
EEG eye states	0.1699	**0.8191**	0.4214	0.3368	0.6856	0.6545	0.9967	0.9992	**1.0**	0.9997
HTRU	0.9059	**0.9966**	0.8922	0.945	0.9922	0.9285	0.9958	0.9971	0.9682	**0.9975**
Letter recognition	0.0008	**0.7975**	0.0828	0.0428	0.148	0.9985	0.9918	0.7563	0.9968	**1.0**
Occupancy	0.6961	**0.9944**	0.5731	0.7138	0.9589	0.7025	0.9968	0.9852	0.9976	**1.0**
Online shopper intention	0.8536	**0.9773**	0.783	0.7997	0.9586	0.1496	**0.9964**	0.3177	0.4988	0.9586
Person activity	0.0308	**0.7478**	0.1	0.0995	0.3056	**1.0**	0.3747	0.5008	0.9995	0.2431
Shuttle	0.1873	**0.9962**	0.1205	0.7751	0.9264	0.8091	0.9997	0.9445	0.9993	**1.0**
Skin	0.9858	**0.9957**	0.3282	0.6987	0.8539	0.0927	**1.0**	0.9204	0.9983	**1.0**
	**Average rule length**					**Number of rules**				
Adult	2.0	16.526	1.0	1.0	4.5556	822	1152	**2**	4	9
Avila	2.0013	17.8939	1.0	1.0	9.0	13483	10769	4	**1**	2
Bank	1.0	12.0694	1.0	1.0	1.0	9	1079	**2**	**2**	**2**
Chess	0.0	16.1055	1.0	1.0	17.7778	0	24769	11	**1**	9
Connect 4	3.0	18.8273	1.0	1.0	4.0	3	7115	**2**	7	9
Cover type	2.0	20.8636	1.0	1.0	3.7778	103187	150534	**2**	18	9
Credit card default	1.6091	14.6388	1.0	1.0	12.3333	8467	2885	**1**	**1**	3
EEG eye states	2.0025	13.8801	1.0	1.0	9.0	16173	2768	2	**1**	2
HTRU	1.0116	9.8545	1.0	1.0	2.0	1382	550	**1**	2	2
Letter recognition	3.0	15.1698	1.0	1.0	13.0	538	13826	5	**1**	4
Occupancy	1.911	9.4584	1.0	1.0	2.0	326	397	2	**1**	2
Online shopper intention	1.9484	11.8254	1.0	1.0	18.0	9623	1346	**2**	**2**	9
Person activity	1.0	18.8819	1.0	1.0	3.3333	33507	91562	**4**	7	9
Shuttle	2.453	17.268	1.0	1.0	6.0	22028	9466	3	**1**	2
Skin	2.0	9.5413	1.0	1.0	2.0	53695	460	2	**1**	2
	**Fraction of classes**					**Fraction overlap**				
Adult	1.0	1.0	0.5	0.5	1.0	0.0236	0.0	1.0	0.4999	0.0
Avila	0.9167	1.0	0.3333	0.0833	0.0833	0.0671	0.0	0.9992	0.0	0.0
Bank	0.5	1.0	0.5	0.5	1.0	0.0013	0.0	1.0	1.0	0.0
Chess	0.0	1.0	0.4444	0.0556	0.2222	0.0	0.0	0.8403	0.0	0.0
Connect 4	0.3333	1.0	0.3333	0.3333	1.0	0.7992	0.0	1.0	0.4286	0.0
Cover type	0.2857	1.0	0.1429	0.1429	0.7143	0.0017	0.0	1.0	0.2816	0.0
Credit card default	1.0	1.0	0.5	0.5	1.0	0.7245	0.0	0.0	0.0	0.0
EEG eye states	1.0	1.0	1.0	0.5	0.5	0.0804	0.0	0.0	0.0	0.0
HTRU	1.0	1.0	0.5	1.0	0.5	0.4095	0.0	0.0	0.1433	0.0
Letter recognition	0.0385	1.0	0.1923	0.0385	0.0385	0.0	0.0	0.7133	0.0	0.0
Occupancy	1.0	1.0	1.0	0.5	0.5	0.0056	0.0	0.0893	0.0	0.0
Online shopper intention	1.0	1.0	1.0	0.5	1.0	0.9781	0.0	0.9397	1.0	0.0
Person activity	0.3636	1.0	0.0909	0.1818	0.2727	0.0	0.0	1.0	0.5696	0.0
Shuttle	1.0	1.0	0.4286	0.1429	0.1429	0.0164	0.0	0.9333	0.0	0.0
Skin	1.0	1.0	1.0	0.5	0.5	0.0	0.0	0.0	0.0	0.0

The scores related to the rule-extraction method(s) that performed the best on each dataset, according to certain metrics, are highlighted in bold to improve the readability of the table. This was not done for all the metrics because in some cases, like completeness, is very clear which method(s) is superior.

Noticeably, C45Rule-PANE and TREPAN produced rulesets that reach 100*%* of completeness throughout all the datasets. This means that their rules cover all the training and test datasets instances. RxREN and RxNCM fail to reach full completeness on four and eight datasets, respectively. REFNE can be ranked as the worst, according to this metric, as its rulesets cover the entire input space on four datasets: Adult, Connect-4, Cover Type, and Online shopper intention. This is because the algorithm can only consider up to three features to create a rule, and, given the size and complexity of the datasets, they might not be enough to cover the entire space. According to the authors who proposed REFNE, each rule should not have more than three antecedents to be comprehensible. Thus they limited to three the number of attributes to create the rule’s antecedents. Furthermore, REFNE fails to produce any rule for the Chess datasets as it cannot find any combination of single values (for the categorical features) or ranges of values (for the continuous features) associated with a single class. The Chess dataset was inspected manually, and it was possible to find only a few combinations of three input features associated with a single class. These candidate rules were identified by the REFNE algorithm but subsequently discarded as they did not reach the fidelity threshold set equal to 50% in this study. This threshold was chosen because it allows REFNE to extract a ruleset for all the other 14 datasets, but it keeps the number of rules relatively low on the majority of them. No other parameter tuning was carried out to avoid the risk of maximising a set of parameters at the expense of others, as this was not the objective of this study. Nonetheless, REFNE generated rulesets with thousands of rules on all the other datasets but four: Bank, Connect 4, Letter recognition and occupancy (notice that the *y*-axis of the barcharts related to the number of rules was converted into the logarithmic scale to make visible the results related to RxNCM, RxREN, and TREPAN which returns rulesets that are significantly smaller than those produced by REFNE and C45Rule-PANE). This places REFNE as the second-worst method in terms of the number of rules, surpassed only by C45Rule-PANE. C45Rule-PANE is the method that produces, by far, the biggest rulesets in terms of the number of rules and average length. However, C45Rule-PANE can be considered the most coherent method as its ruleset cover all the output classes without overlapping areas. Apparently, TREPAN is the method that manages to reach the best compromise between syntactic simplicity and coherence as it generates small rulesets containing rules that do not overlap. However, they manage to cover all the output classes on five datasets: Adult, Bank, Connect 4, Credit card default, and Online shopper intention. RxNCM and RxREN are the methods that extract the smallest rulesets, but they cannot be considered coherent, especially in terms of overlapping areas. This is because their algorithms analyse each input feature separately. They split each feature into intervals, according to the distribution output class within those intervals, in isolation, without considering the interactions between features.

Focusing on the four metrics assessing the degree of agreement between the rule extractor and the underlying model (completeness, correctness, fidelity, and robustness), it is possible to notice that REFNE can be still ranked as the worst also in terms of correctness and fidelity. On the other hand, C4.5Rule-PANE performed better than the other four methods for explainability in terms of correctness and fidelity whilst has mixed results in terms of robustness. However, as noticed before, the barcharts related to the number of rules and their average length suggest some drawbacks. To reach these results, C4.5Rule-PANE indeed produced the biggest rulesets across all the fifteen datasets. This hampers the interpretability of its rulesets. TREPAN does not excel in any of these four metrics, except completeness, but it has better results than RxNCM, RxREN, and REFNE regarding correctness and fidelity. On the other hand, TREPAN seems, in general, less robust than these three methods. Likely, this is because their algorithms are forced to generate small rulesets that must cover ample areas of the input space; thus, small perturbations of the input data go unnoticed.

**FIGURE 9:**
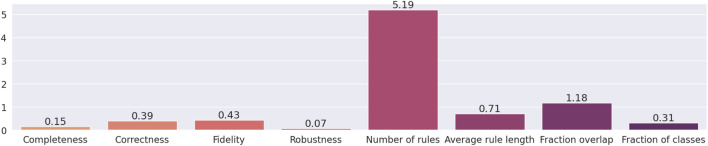
Information entropy of the eight metrics for explainability.

Finally, the information entropy of the metrics was calculated to quantify the level of “information” or “uncertainty” inherent in their possible outcomes (see Figure 9). The entropy was assessed with the Kullback-Leibler divergence, as implemented in the Python package Scipy, which is equal to *∑*_*n*_ {*p*_*k*_ (*n*)* log [*p*_*k*_ (*n*)/*q*_*k*_ (*n*)]} where *p*_*k*_ (*n*) and *q*_*k*_ (*n*) are the probability and the numeric value of event *n*, respectively. The events correspond to the values of the eight metrics across the datasets and the rule extractors. All the events have a probability equal to one. The number of rules is the metric with the highest entropy, followed by fraction overlap. This is to be expected as these are the two metrics with the highest variability in their results. The number of rules can go from 1 up to several thousand. There are two methods, C4.5Rule-PANE and TREPAN, that generate ruleset without overlapping rules, whilst other methods can have rules that cover the same input space, thus bringing the fraction overlap up 100%.

**FIGURE 10:**
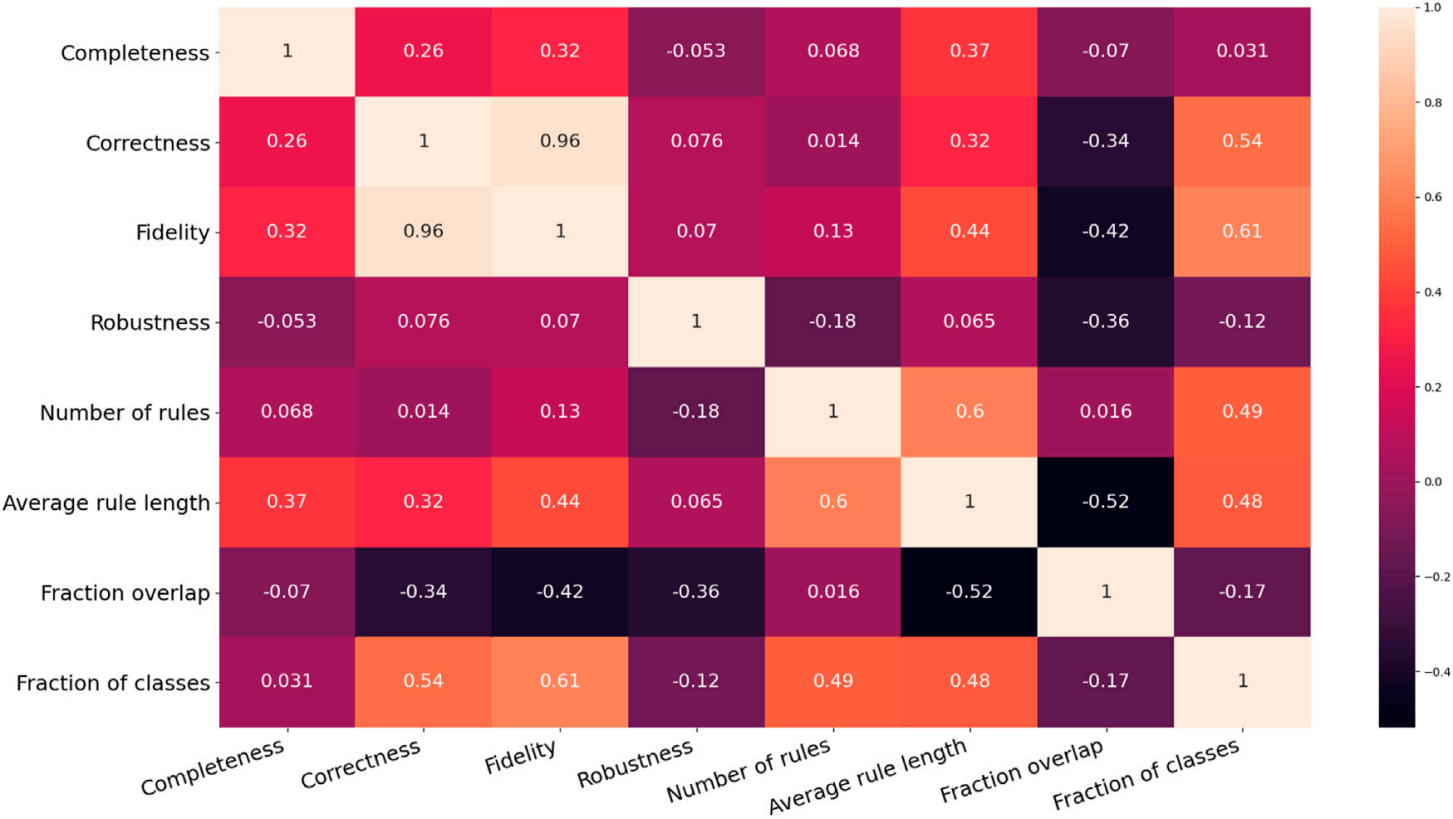
Correlation matrix of the results related to the eight evaluation metrics exploited to assess the degree of explainability of machine-generated rulesets.

In summary, this experiment provided a few interesting insights into the analysed rule-extraction methods. Firstly, the metric number of rules have an entropy that is, considerably higher than the entropy of the other metrics, thus making it the most informative metric of the whole bunch. Secondly, the results suggest a trade-off between the size of the rulesets in terms of both the number of rules and antecedents and the other six metrics, namely completeness, correctness, fidelity and robustness, the fraction of classes and overlap. In other words, when a method for rule extraction produces small rulesets or short rules, then the latter six metrics tend to score low. To test this hypothesis, the rank Spearman’s rank correlation coefficients between each pair of metrics were calculated (see Figure 10). The range of these coefficients was split into the three subsets to analyse the correlations of the input features of the 15 datasets. One pair of variables can be considered strongly correlated since its Spearman’s rank coefficient is higher than 0.66: fidelity-correctness. The pair fidelity-fraction of classes has a coefficient of 0.61, just below the threshold. This result should be expected as fidelity cannot be attained if the rulesets ignore the minority classes. However, fidelity-correctness is not intuitive as a ruleset cannot be both faithful and correct if the underlying model reaches a low prediction accuracy. This happened because 12 out of 15 of the neural networks trained on the chosen datasets assign more than 70% of the input instances to the correct class. Additionally, all the five rule-extraction methods mimic as much as possible the inferences made by the underlying neural networks that were structured to reach the highest feasible prediction accuracy. Consequently, the most faithful methods also reach a high level of correctness. There are other pairs of metrics that can be considered moderately correlated, such as completeness-average rule length and average rule length-fidelity, as they fall in the range (0.33, 0.66). Noticeably, the number of rules and average rule length are moderately correlated with all the other metrics and also among them (their Spearman’s rank correlation coefficient is 0.6). This means that there is no strong evidence supporting our observation that there is a trade-off between the size of the rulesets and the other six metrics. It is worth pointing out that fraction overlap shows negative correlations with all the other metrics (the only exception is the number of rules), whereas all the other pairs are positively correlated, with a few exceptions such as robustness-completeness and robustness-number of rules. However, none of these can be considered a strong correlation, and this might be the outcome of the specific combinations of data, machine-learned models and rule-extractors analysed in this experiment.

**FIGURE 11:**
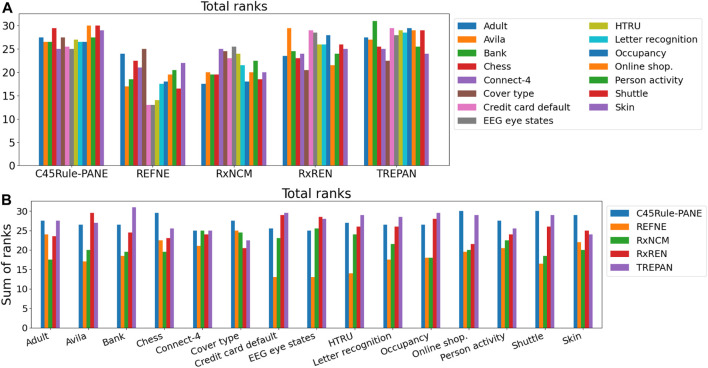
Total ranks of the five rule-extraction methods, grouped by methods **(A)** and datasets **(B)**.

**FIGURE 12:**
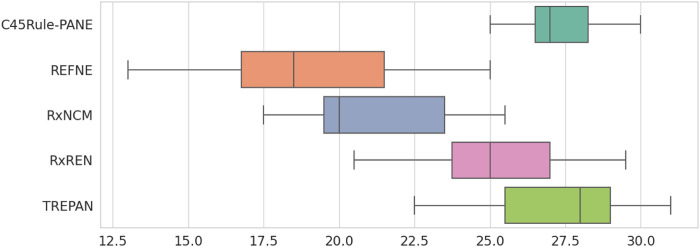
Distribution of the total ranks of the five rule-extraction methods across datasets and metrics.

Finally, the Friedman test was applied to check whether a method can be considered superior (or inferior) to the others according to the eight metrics under analysis across the selected datasets. The test output statistics and *p*-values are reported in Table 5 and Figure 11. None of the *p*-values is lower than the typical tolerance level of 1%, and only one is lower than 5% (Credit card default). Thus, there is no evidence supporting the alternative hypothesis for 14 out of 15 datasets, meaning that none of the five methods performs consistently better (or worst) than the others. In the case of the Credit card default, there is weak evidence that a method ranks consistently higher or lower than the others. Looking at Figure 11, it is possible to notice that REFNE’s total ranks are significantly lower than the ranks of the other methods; thus, it might be considered the worst method. The Credit card default dataset has no categorical features and only two output classes; thus REFNE struggles in finding combinations of values of the input features associated with a class. The EEG eye state dataset is similar to the Credit Card default and, as a matter of fact, its *p*-value is 6% and it is the second lowest one. The Friedman test was also applied by considering the datasets as repetitions of the same experiment, thus calculating a unique *p*-value to determine if a rule-extraction method performs better (or worse) than the others across all the datasets. The result is a Friedman coefficient of −1414 and a *p*-value equal to 1. A post-hoc analysis revealed that the distributions of the total ranks of the five methods across all the metrics and datasets overlap (Figure 12). This supports the outcome of the Friedman test because none of the methods ranks consistently as first (or last). In conclusion, it is not possible to say that a method is always superior to the others. There is not a one-fits-all solution. Despite this finding, the eight evaluation metrics provide a quick and objective tool to highlight the strengths and weaknesses of each method for explainability. This is vital information for researchers when choosing the most suitable explanation tool for their projects.

**TABLE 5:** Output statistics and *p*-values of the Friedman test, grouped by dataset.

	Dataset list				
	Adult	Avila	Bank	Chess	Connect-4
Test statistic	4.0	6.139	6.574	3.090	0.716
*p*-value	0.406	0.189	0.16	0.543	0.949
Superior/inferior method	NA	NA	NA	NA	NA
	**Cover type**	**Credit card default**	**EEG eye state**	**HTRU**	**Letter recognition**
Test statistic	1.623	10.482	9.042	7.614	4.482
*p*-value	0.805	**0.033**	0.060	0.107	0.345
Superior/inferior method	NA	REFNE (inferior)	NA	NA	NA
	**Occupancy**	**Online shopper intention**	**Person activity**	**Shuttle**	**Skin**
Test statistic	6.730	5.790	1.506	7.974	2.809
*p*-value	0.151	0.215	0.825	0.093	0.590
Superior/inferior method	NA	NA	NA	NA	NA

The bold value highlights the unique case where the Friedman test returns a p-value lower than 5%, thus providing evidence that there is a rule-extraction method which can be ranked as the best (or the worst).

## 5 Final Remarks and Recommendations

This study presented a novel approach to evaluate and compare five methods for explainability, namely C45Rule-PANE, REFNE, RxNCM, RxREN, and TREPAN, which extract rules from black-box machine-learned models based on feed-forward neural networks. These models were trained on 15 datasets with handcrafted features that humans manually engineered. Missing data and strongly correlated variables were removed from the datasets in advance of the training of the model to avoid issues during the learning process. Additionally, the distribution of the input instances over the various output classes was taken into consideration to ensure that the training instances were evenly spread among the target classes. The split into the training and validation datasets was carried out with the stratified five-fold cross-validation technique to ensure the original proportion of each class was preserved. The SMOTE algorithm was applied to each fold to up-sample the minority classes and guaranteeing that the classifiers did not ignore them. The surrogate model, out of five, with the highest validation accuracy, was selected as the final model. Eight quantitative and objective metrics were identified in the literature to assess the degree of explainability of the rulesets extracted by the five XAI methods from the 15 trained neural networks. These are ruleset cardinality, number of antecedents, completeness, fidelity, correctness, robustness, and fraction of classes and overlap. Eventually, the Friedman test verified whether one of the selected methods ranked consistently higher than the others across these metrics. The experiment did not provide sufficient evidence to support the alternate hypothesis of the Friedman test. Hence, none of the methods outperformed the others throughout all the datasets. However, the results suggested the presence of a trade-off between the two metrics measuring the syntactic simplicity of the rulesets (number of rules and average length) and the other six metrics. A correlation analysis was carried out over the eight metrics to support this observation, but only fidelity and correctness showed a strong correlation. At the same time, the number of rules and the average rule length were weakly correlated with the other six metrics. Thus, the observation that there is a trade-off between these two groups of metrics had to be rejected. Despite all, the selected metrics proved to be apt to highlight the weaknesses and strengths of the tested rule-extraction methods, thus providing scholars with a multi-dimensional approach to evaluate their XAI methods in a straightforward, quantitative, and objective manner without any human intervention and bias. To the best of our knowledge, this is the first study of its kind that tested many rule-extraction methods for explainability over such a variety of datasets, designed for various classification problems, with metrics that measure different aspects of machine-generated rulesets, such as their syntactic simplicity, their internal coherence and their degree of agreement with the underlying models.

Future work will extend this research study by investigating additional explainability metrics, by training deeper neural networks, by employing datasets containing additional types of input data, such as texts and images. Furthermore, with a human-in-the-loop approach, it is worth investigating the correlation of selected quantitative objective metrics with subjective perceptions of humans. In this regard, developing a valid and reliable questionnaire for explainability represents the next challenge for the XAI community. Another future work is to add an extra layer of explainability that transforms the machine-generated rules into a format, such as textual or visual explanations, which is more appealing for humans, in particular lay end-users. A way to produce this layer can consist of exploiting advances in defeasible reasoning and argumentation by using knowledge-bases constructed with a human-in-the-loop approach (Rizzo and Longo, 2018b,a; Zeng et al., 2018). Another possible solution is to utilise neuro-symbolic learning and reasoning in parallel, each one informing the other at all stages of model construction and evaluation (Garcez et al., 2015).

## Data Availability

Publicly available datasets were analyzed in this study. This data can be found here: https://archive.ics.uci.edu/ml/datasets.php.

## Footnotes

1
https://archive.ics.uci.edu/ml/index.php
