# Supplementary material for: A Quantitative Evaluation of Global, Rule-Based Explanations of Post-Hoc, Model Agnostic Methods
Source: Front Artif Intell. 2021 Nov 3;4:717899. doi: 10.3389/frai.2021.717899 (PMC8596373; doi:10.3389/frai.2021.717899)
Supplement: Supplementary file 1 [file DataSheet1.pdf]

Supplementary Material

0.1 Algorithms of rule-extraction methods

```
1: REFNE(X):
2:      R = empty ruleset
3:      Create synthetic dataset S by varying each input feature of X
            across its value range
4:      y' = Oracle(model, S)
5:      Select a categorical feature F of S
6:      Try to find value U such that all y' with U belong to class C
7:      If U is found:
7:          Create rule r
8:      Else:
9:          If not all categorical feature have been examined and
                the rule has less than three antecedents:
10:             Select categorical feature G
11:             Try to find value V of G such that all the y' with V belong to class C
12:             If V is found:
13:                 Create rule r
14:             Else:
15:                 Go to step 9
16:         Else:
17:             Discretize continuous variables with ChiMerge and go
                to step 6
8:      If fidelity of r > delta:
9:          Add r to ruleset R
10:         Remove y' covered by r from S
11:         If size(S) = 0 return R
```

Figure S1. Algorithm of REFNE

```
1:  C4.5Rule-PANE(X):
2:     y' = Oracle(model, X)
3:     Create synthetic dataset S by varying each input feature of X across
            its value range
4:     y'' = Oracle(model, S)
5:     xSynth = concatenate X and S
6:     ySynth = concatenate y' and y''
7:     C45_build_tree(xSynth, ySynth)
```

Figure S2. Algorithm of C4.5Rule-PANE

```
1: TREPAN(training_examples, features):
2:      Queue = 0
3:      For each example E in training_examples:
            class_E = Oracle(model, E)
4:      Initialize the root of tree T as leaf node
5:      Put (T,training_examples,{}) into Queue
6:      While size(Queue) > 0 & size(T) < tree_size_limit:
7:          Remove node N from head of Queue
8:          example_N = example set stored with N
9:          constraint_N = constraint set stored with N
10:         Use features to build set of candidate splits
11:         Use example_N and calls to Oracle(model, constrains_N) to evaluate splits
13:         S = best binary split
14:         Search for best m-of-n split S’ using S as seed
15:         Make N an internal node with split S’
16:         For each outcome s of S’
17:             Make C a new child node of N
18:             constraints_C = constraints_N AND {S’ = s}
19:             Use calls to Oracle(model, constraints_C) to determine
                    if C should remain a leaf
20:             Otherwise
21:                 examples_C = members of examples_N with outcome s on split S’
22:                 Put (C,example_C,constraint_C) into Queue
23:     Return T
```

Figure S3. Algorithm of TREPAN

```
1: RxREN(X):
2:      T = set of correctly classified instances of X
3:      original_acc = model accuracy
4:      For each input feature F:
5:          Remove F and estimate new accuracy n_acc
6:          E_F = set of incorrectly classified instances of T by pruned
                network without F
7:          err_F = cardinality of E_F
8:      Estimate the new accuracy of pruned network n_acc
9:      If n_acc > original_acc - 1%,
10:         Prune feature with error = min(err_F for every F)
11:         Go back to step 4
12:     For each feature F of the pruned network:
13:         Group examples belonging to E_F with respect to target class C_k
            and find number of instances q_Fk
14:         Select classes with q_Fk > alpha * err_F with alpha in [0.1, 0.5]
15:         Find minimum L_Fk and maximum U_Fk value of instances from E
                belonging to C_k if selected
16:     Construct rules for each selected class C_k using L_Fk and U_Fk as
            rule antecedents
17:     Check if each new rule improve the accuracy of the entire ruleset
18:     Classify test examples using ruleset
19:     Find min and max of misclassified examples corresponding
            to each class of each feature of the pruned network
20:     Replace previous data ranges if new min and max improves
            accuracy of ruleset
```

Figure S4. Algorithm of RxREN

```
1: RxNCM(X):
2:      T = set of correctly classified instances of X
3:      original_acc = model accuracy
4:      For each input feature F:
5:          Remove F and estimate new accuracy n_acc
6:          E_F = set of incorrectly classified instances of T by pruned
                network without F
7:          err_F = cardinality of E_F
8:      Estimate the new accuracy of pruned network n_acc
9:      If n_acc > original_acc,
10:         Prune feature with error = min(err_F for every F)
11:         Go back to step 4
12:     For each feature F of the pruned network:
13:         Find the set of instances P_F of T properly classified by the pruned network
14:         Group examples belonging to E_F and P_F with respect to target class C_k
            and find number of instances q_Fk
15:         Select classes with q_Fk > alpha * mp_F with alpha in [0.1, 0.5]
                where mp_F is the cardinality of E_F and P_F
16:         Find minimum L_Fk and maximum U_Fk value of instances from E
                belonging to C_k if selected
17:     Construct rules for each selected class C_k using L_Fk and U_Fk as
            rule antecedents
18:     Check if each new rule improve the accuracy of the entire ruleset
19:     Classify test examples using ruleset
20:     Find min and max of properly classified and misclassified examples
            corresponding to each class of each feature of the pruned network
21:     Replace previous data ranges if new min and max improves
            accuracy of ruleset
```

Figure S5. Algorithm of RxNCM

0.2 Modification of the REFNE algorithm

The REFNE method, as designed by its authors, requires to discretise the continuous variables by using a modified version of the ChiMerge algorithm which is a supervised, bottom-up data discretization approach. The input instances are sorted according to the value of the continuous variable to be discretised and each value is considered a separate cluster. Subsequently, at each iteration, the statistical measure χ^2 of every pair of adjacent clusters is computed and those with the lowest χ^2 value are merged. The χ^2 value of a pair of clusters is calculated with the following formula:

$$\chi^2 = \sum_{i=1}^{2} \sum_{j=1}^{k} \frac{(A_{ij} - E_{ij})^2}{E_{ij}}$$

where k is the number of classes, A_{ij} is the number of samples in the ith cluster that belongs to the jth class, E_{ij} is the expected frequency of A_{ij}.

In the original ChiMerge approach, the process ends when the χ^2 of all the pairs of clusters exceeds a user-defined threshold which correspond to consider all the adjacent clusters significantly different according to the χ^2 independence test. In REFNE, the process ends when it is not possible to merge clusters that contain instances belonging to the same output class. This means, in mathematical terms, fixing the threshold to zero as A_{ij} is equal to E_{ij} when the pair of clusters to be merged contains samples belonging to the same class. In this case, the ChiMerge algorithm stops at the first iteration as χ^2 cannot be negative by definition. However, this corresponds to grouping adjacent samples that belong to the same class, which is computationally less onerous than the calculation of the χ^2 statistics which is superfluous. An example can be given by analysing the Iris dataset that was also used by the authors to show how ChiMerge works. In the original study Kerber (1992), the data are clustered by the variable 'sepal length' (the distribution of the data is shown in Figure S6) an the threshold is set to 4.6 which corresponds to the 90% significance level. Table S1 reports the final discretization of the sepal length variable.

Table S1. Output of the original version of the ChiMerge discretization algorithm applied to the sepal length variable of the Iris dataset.

Interval	Frequency Setosa	Frequency Versicolor	Frequency Virginica	χ^2
4.3 - 5.4	45	6	1	30.9
5.5 - 5.7	4	15	2	6.7
5.8 - 6.2	1	15	10	4.9
6.3 - 7.0	0	14	25	5.9
7.1 - 7.9	0	0	12	

In the REFNE version, the same discretization process returns the list of clusters, reported in Table S2, that contain samples belonging to the same class. Some values are associated with multiple classes, so they cannot be merged with other adjacent values and must be considered as standalone clusters. This discretization has been carried out with both the ChiMerge algorithm and by performing a simple merge of adjacent values belonging to the same class. The latter algorithm was 5 times faster than ChiMerge.

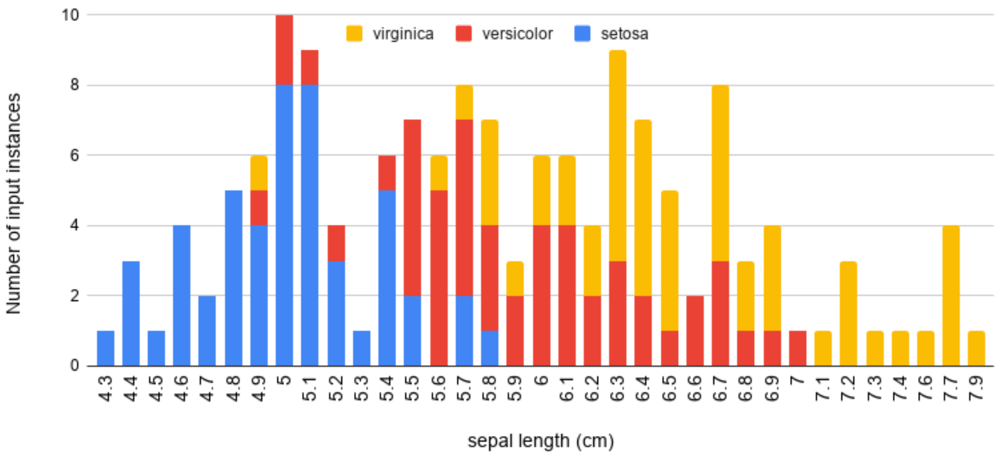

Figure S6. Distribution of the number of input instances, split by output class, of the Iris dataset sorted by the value of the 'sepal length' variable.

Table S2. Output of the REFNE version of the ChiMerge discretization algorithm applied to the sepal length variable of the Iris dataset.

Interval	Frequency Setosa	Frequency Versicolor	Frequency Virginica
4.3 - 4.8	16	0	0
4.9	4	1	1
5.0	8	2	0
5.1	8	1	0
5.2	3	1	0
5.3	1	0	0
5.4	5	1	0
5.5	2	5	0
5.6	0	5	1
5.7	2	5	1
5.8	1	3	3
5.9	0	2	1
6.0	0	4	2
6.1	0	4	2
6.2	0	2	2
6.3	0	3	6
6.4	0	2	5
6.5	0	1	4
6.6	0	2	0
6.7	0	3	5
6.8	0	1	2
6.9	0	1	3
7.0	0	1	0
7.1 - 7.9	0	0	12

REFERENCES

Kerber, R. (1992). Chimerge: Discretization of numeric attributes. In *Proceedings of the tenth national conference on Artificial intelligence*. 123–128
